# Effects of three-dimensional soil heterogeneity and species composition on plant biomass and biomass allocation of grass-mixtures

**DOI:** 10.1093/aobpla/plab033

**Published:** 2021-05-28

**Authors:** Yongjie Liu, Guoe Li, Mingxia Wang, Wenjing Yan, Fujiang Hou

**Affiliations:** State Key Laboratory of Grassland Agro-ecosystems; Key Laboratory of Grassland Livestock Industry Innovation, Ministry of Agriculture and Rural Affairs; College of Pastoral Agriculture Science and Technology, Lanzhou University, Lanzhou 730020, China

**Keywords:** Biomass allocation, patch size, soil heterogeneity, species composition, three dimensions

## Abstract

Soil heterogeneity significantly affects plant dynamics such as plant growth and biomass. Most studies developed soil heterogeneity in two dimensions, i.e. either horizontally or vertically. However, soil heterogeneity in natural ecosystems varies both horizontally and vertically, i.e. in three dimensions. Previous studies on plant biomass and biomass allocation rarely considered the joint effects of soil heterogeneity and species composition. Thus, to investigate such joint effects on plant biomass and biomass allocation, a controlled experiment was conducted, where three levels of soil heterogeneity and seven types of species compositions were applied. Such soil heterogeneity was developed by filling nutrient-rich and nutrient-poor substrates in an alternative pattern in pots with different patch sizes (small, medium or large), and species compositions was achieved by applying three plant species (i.e. *Festuca elata*, *Bromus inermis*, *Elymus breviaristatus*) in all possible combinations (growing either in monoculture or in mixtures). Results showed that patch size significantly impacted plant biomass and biomass allocation, which differed among plant species. Specially, at the pot scale, with increasing patch size, shoot biomass decreased, while root biomass and R:S ratio increased, and total biomass tended to show a unimodal pattern, where the medium patch supported higher total biomass. Moreover, at the substrate scale, more shoot biomass and total biomass were found in nutrient-rich substrate. Furthermore, at the community scale, two of the three target plant species growing in monoculture had more shoot biomass than those growing together with other species. Thus, our results indicate soil heterogeneity significantly affected plant biomass and biomass allocation, which differ among plant species, though more research is needed on the generalization on biomass allocation. We propose that soil heterogeneity should be considered more explicitly in studies with more species in long-term experiments.

## Teaser

Soil heterogeneity significantly affects plant dynamics such as plant growth and biomass. A controlled experiment was conducted to explore the effects of three-dimensional soil heterogeneity and species composition on plant biomass allocation, where such soil heterogeneity was developed by filling nutrient-rich and nutrient-poor substrates in an alternative pattern in pots with different patch sizes (small, medium or large), and species compositions was achieved by applying three plant species in all possible combinations. Our study demonstrated that soil heterogeneity significantly impacted plant biomass allocation, which differed among plant species.

## Introduction

Soil heterogeneity refers to the un-uniform distribution pattern of soil resources (e.g. nutrient, water and microbe). It includes two components, i.e. qualitative and configurational components. The former reflects differences in soil nutrients, moisture, microbes etc. between locations in the soil (Maestre and Cortina 2002; Reynolds and Haubensak 2009; Feeser *et al*. 2018), while the latter refers to the patch size of these locations (Dufour *et al*. 2006). Soil heterogeneity as a key characteristic of soils (Fitter *et al*. 2000; Huber-Sannwald and Jackson 2001; Hutchings *et al*. 2003) significantly affects plant dynamics such as root distribution (Liu *et al*. 2017a) and plant biomass (Baer *et al*. 2003; Maestre and Reynolds 2007; Liu *et al*. 2017b). Soil heterogeneity varies both horizontally and vertically (Stewart *et al*. 2000), while most studies developed soil heterogeneity in two dimensions, i.e. either horizontally or vertically (Maestre and Reynolds 2006; Williams and Houseman 2014; Wubs and Bezemer 2016). However, soil heterogeneity in natural ecosystems varies in three dimensions, i.e. both horizontally and vertically, while only a few studies have explored such effects on plants (Liu *et al*. 2017a, b, 2019; Liu and Hou 2021). Note that soil heterogeneity in this study refers to configurational heterogeneity.

Soil heterogeneity significantly affected plant biomass, and previous studies found that soil heterogeneity tends to improve plant biomass via promoting nutrient capture (Mommer *et al*. 2012) or increasing resources absorption from nearby patches (Liu *et al*. 2017a). Relative higher soil heterogeneity could support higher plant diversity (Liu *et al*. 2019). Moreover, a community including more species tends to have higher biomass (Wang *et al*. 2008; Xue *et al*. 2016). Therefore, higher soil heterogeneity is expected to support more biomass. This could be derived from complementarity effects (Hector 1998; Loreau and Hector 2001; Bai *et al*. 2004), which refers to that plants from different species having different traits such as different root geometries, and they could occupy totally different spaces, fulfilling complementary functional roles (Loreau 2000). It includes two main effects, i.e. niche differentiation (i.e. increasing resource-use efficiency, Tilman *et al*. 2001) and facilitation (i.e. positive interaction between species, Ledgard and Steele 1992; Liu *et al*. 2016). Biomass allocation is crucial for understanding the plant dynamics and structure and functioning of plant communities (Poorter *et al*. 2012). However, these studies did not clearly consider the joint effect of soil heterogeneity and species composition (Maestre *et al*. 2006; García-Palacios *et al*. 2012).

To explore the effect of three-dimensional soil heterogeneity and species composition on plant biomass and biomass allocation, a controlled experiment was conducted, where three levels of three-dimensional soil heterogeneity and seven types of species compositions were applied. Such three-dimensional soil heterogeneity was developed by filling nutrient-rich and nutrient-poor substrates in pots with different patch sizes (small, medium or large) in an alternative pattern in all dimensions (i.e. both horizontally and vertically). In other words, each substrate was surrounded by a different substrate both in horizontal and vertical directions in each pot. Species compositions were achieved by applying three plant species (*Festuca elata*, *Bromus inermis* and *Elymus breviaristatus*, labelled as FE, BI and EB, respectively) in all possible combinations (i.e. FE, BI, EB, FE+BI, FE+EB, BI+EB, FE+BI+EB). Specially, our hypotheses are: (i) At the pot scale, pots with smaller patch are assumed to have more biomass since plants growing there could easier access to resource from neighbouring patches (Liu *et al*. 2017a, b), which may differ among grass species. (ii) At the substrate scale, differences of biomass between nutrient-rich and nutrient-poor substrate should be smaller in pots with smaller patch, where plants could easier uptake resources from their neighbouring patch due to the short distance in pots with smaller patch (Liu *et al*. 2017a). (iii) At the community scale, pots with more species would support more biomass than those with fewer species due to the complementarity effects, where complementary species in plant communities could better use resources than in monoculture (Loreau and Hector 2001; Bai *et al*. 2004).

## Materials and Methods

### Field site

A manipulative experiment was conducted from 5 June to 6 September 2020 at Linze Grassland Agriculture Trail Station of Lanzhou University (100°3′25ʺE, 39°14′30″N). This station is in the middle of Hexi Corridor with an average altitude of 1400 m (Hou and Shen 1999). The local climate is characterized by temperate continental arid monsoon climate, where mean annual temperature and mean annual precipitation are 9.3 °C and 112.9 mm, respectively. Temperature varies from −28 to 38 °C. More than 60 % of the rainfall occurs in summer and autumn. The local soils are salinized meadow soil and saline soils (Zhu *et al*. 1997), which is caused by the large difference of low rainfall and the high evaporation.

**Figure 1.**
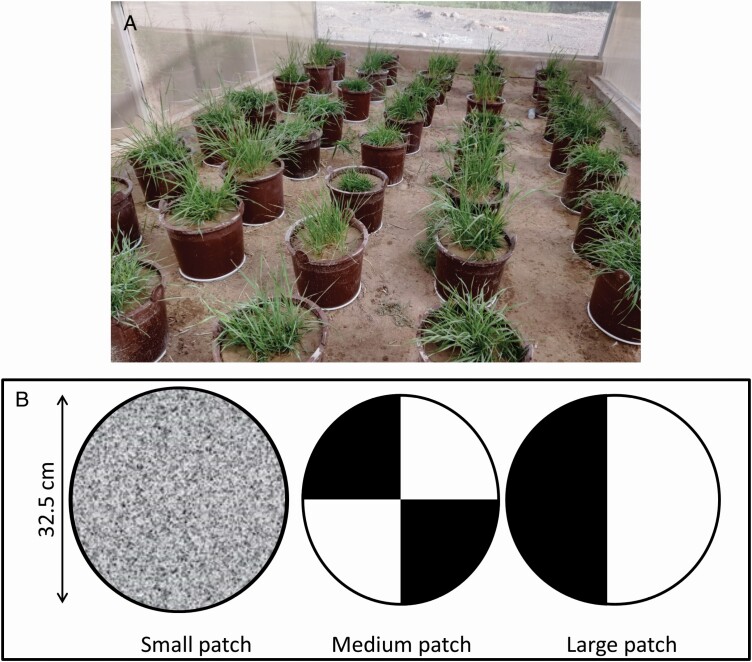
Set-up of the experiment. All pots were set in four separated net rooms, which have the same condition. Each net room is treated as a replication (A), including pots with three patch sizes (small, medium and large, B) and seven species combinations (*Festuca elata* (FE), *Bromus inermis* (BI), *Elymus breviaristatus* (EB), FE+BI, FE+EB, BI+EB and FE+BI+EB).

To explore the joint effects of soil heterogeneity and species composition on plant biomass, three levels of soil heterogeneity and seven types of species compositions were applied. Here soil heterogeneity was developed by filled two types of substrates (nutrient-rich and nutrient-poor substrates) in pots with different patch sizes (small, medium and large patch) in an alternative pattern in all dimensions (Fig. 1). Soil heterogeneity to some extent increases with decreasing patch size (Li and Reynolds 1995; Maestre and Reynolds 2006). Nutrient-rich and nutrient-poor substrates were created by mixing local soil and sand in the ratio of 8:2 and 2:8, respectively. The local soil was collected from local crop field, and the sand was bought from a local commercial company. Note that these two substrates differ not only in nutrients, but also in other traits such as water holding capacity, pH and microbes. They are named as ‘nutrient-rich’ and ‘nutrient-poor’ substrate for simplification. Three target grasses (i.e. *F. elata*, *B. inermis* and *E. breviaristatus*, named as FE, BI and EB, respectively) were used to create different species compositions, resulting in seven types of species compositions (i.e. FE, BI, EB, FE+BI, FE+EB, BI+EB, FE+BI+EB). Same number of seeds was added into all pots at the beginning of the experiment to reduce the effect of plant density.

Pots with average 29.3 cm diameter (32.5 cm top diameter, 26.0 cm bottom diameter) and 34.5 cm height were used in this experiment. All plants were grown in these pots with the three patch sizes. Each treatment had four replications. There were five holes with 10-mm-diameter holes in the bottom of each pot ensured the drainage of water. During the experiment, the same amount of water was added into each pot once per week to remove the effect of water amount on plant biomass (Liu and Li 2020). Water was added evenly through using a hose with shower by hand to keep water at the soil surface. All pots were evenly distributed in the four identical net rooms, and results showed no significant effect of net room on shoot biomass, root biomass, total biomass and R:S ratio among them (*P* = 0.469, 0.428, 0.659 and 0.377 for these parameters, respectively, analyses were done with MANOVA).

Shoots and roots of grasses growing in each pot were harvested at the end of the experiment, and roots were carefully washed out from their growing soil. They were over-dried at 65 °C to constant weight and weighted.

### Statistical analyses

In order to explore the drivers of plant biomass and biomass allocation from large to small scales, analyses were conducted at the pot, substrate and community scales. Plant density was measured only at the pot scale, thus density was treated as a random only at this scale. **At the pot scale**, linear mixed model (LMM) was conducted to explore effects of patch size, species composition and their interactions on shoot biomass, root biomass, total biomass and root:shoot ratio of plants growing in all the three patch sizes, where plant density was treated as a random factor. **At the substrate scale**, MANOVA was conducted to explore effects of patch size, soil type, species composition and their interactions on shoot biomass, root biomass, total biomass and root:shoot ratio of plants growing in pots with medium and large patch, but not in small patch as soil type cannot be separated in this case. **At the community scale**, one-way ANOVA was done to investigate effects of species composition on shoot biomass. Moreover, to explore the effects of patch size, soil type, species composition, species and their interactions on shoot biomass of plants growing in pots with medium and large patch, another MANOVA was conducted only for pots with medium and large patch sizes, likewise not in small patch since soil type cannot be separated in pots with small patch. Results showed that species significant affected shoot biomass, thus each species was analysed separately, where MANOVA was conducted to explore effects of patch size, soil type, species composition and their interactions on shoot biomass of *F. elata*, *B. inermis* and *E. breviaristatus*. *Post hoc* analyses (pairwise comparisons with Bonferroni corrections) were done to compare the differences among variables. Note that LSD and Tukey corrections were conducted as well, which led to the same results with Bonferroni corrections in regard of significant and non-significant difference. Log-transformations were done when necessary. All statistics were done with SPSS 21.0.

## Results

**Figure 2.**
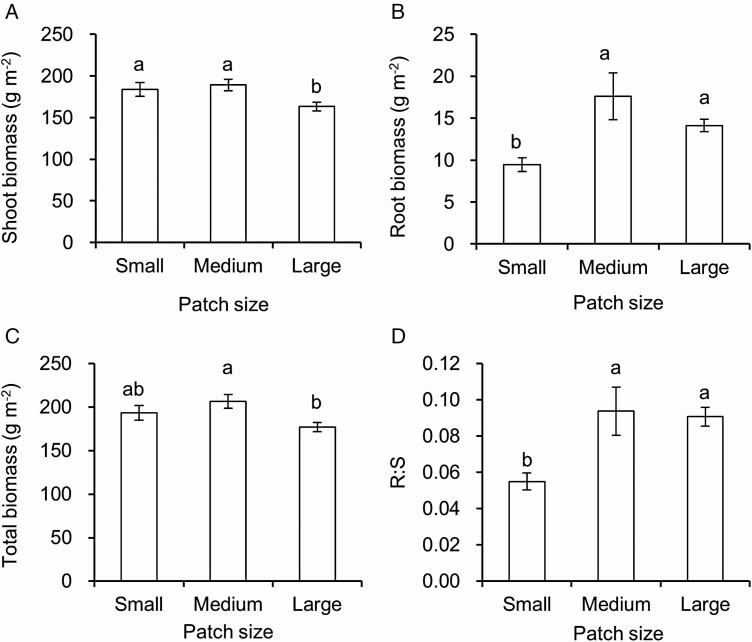
At the pot scale, mean ± SE of shoot biomass (A), root biomass (B), total biomass (C) and root:shoot ratio (R:S, D) along all the three patch sizes. Significant differences between treatments are labelled by different letters (*post hoc* analysis with Bonferroni corrections).

**At the pot scale**, patch size and species composition significantly affected shoot biomass, root biomass, total biomass and R:S (Table 1). Specifically, shoot biomass decreased with increasing patch size (Fig. 2A), while root biomass (Fig. 2B) and R:S (Fig. 2D) increased with increasing patch size. These tended to be a unimodal pattern between patch size and total biomass, where it firstly increased and then decreased after reaching a peak (Fig. 2C).

**Table 1.** At the pot scale, effects of patch size (small, medium and large), species composition and their interaction on shoot biomass, root biomass, total biomass and root:shoot ratio of grasses in pots with small, medium and large patch in LMM, where degree of freedom (df), *F*-values and *P*-value are given, and significant results (*P* < 0.05) are labelled in bold.

	Shoot biomass			Root biomass			Total biomass			Root:shoot ratio		
	df	*F*	*P*	df	*F*	*P*	df	*F*	*P*	df	*F*	*P*
Patch size	2,143	4.5	**0.013**	2,143	12.3	**<0.001**	2,143	4.1	**0.019**	2,143	18.9	**<0.001**
Species composition	6,143	5.0	**<0.001**	6,143	4.6	**<0.001**	6,143	4.2	**0.001**	6,143	9.5	**<0.001**
Patch size × Species composition	12,143	0.4	0.944	12,143	0.6	0.840	12,143	0.4	0.964	12,143	0.8	0.476

**Figure 3.**
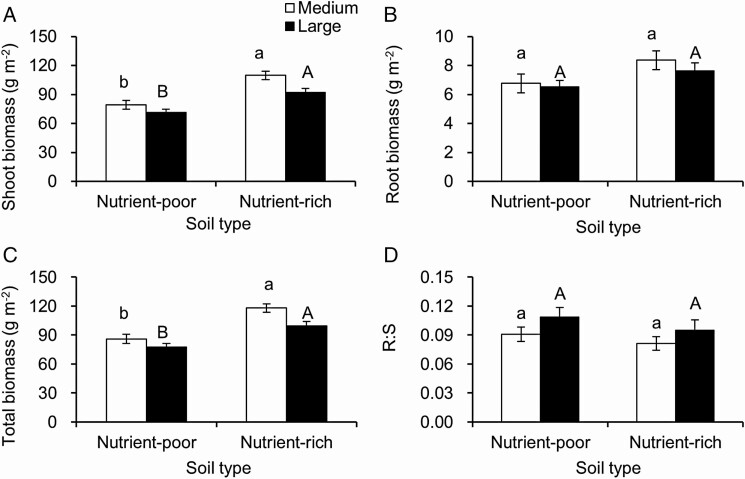
At the substrate scale, mean ± SE of shoot biomass (A), root biomass (B), total biomass (C) and root:shoot ratio (R:S, D) along soil type (nutrient-rich or nutrient-poor substrate), separated by patch size (only for medium and large patch, and small patch is not considered since soil type cannot be separated in this case). Significant differences are labelled by different letters (*post hoc* analysis with Bonferroni corrections).

**At the substrate scale**, species composition significantly affected shoot biomass, root biomass, total biomass and R:S (Table 2). Soil type significantly affected shoot biomass, root biomass and total biomass (Table 2). Patch size significantly affected shoot biomass and total biomass (Table 2). Comparing with nutrient-poor substrate, nutrient-rich substrate supported more shoot biomass (Fig. 3A) and total biomass (Fig. 3C), while no differences were found in root biomass (Fig. 3B) and R:S (Fig. 3D) between nutrient-rich and nutrient-poor substrate. These patterns were found both in pots with medium patch and large patch sizes.

**Table 2.** At the substrate scale, effects of patch size (medium and large), soil type (nutrient-rich and nutrient-poor substrate), species composition and their interactions on shoot biomass, root biomass, total biomass and root:shoot ratio of grasses in MANOVA, where degree of freedom (df), *F*-values and *P*-value are given, and significant results (*P* < 0.05) are labelled in bold. Note that this analysis was done only for pots with medium and large patch as soil type cannot be separated in pots with small patch.

	Shoot biomass			Root biomass			Total biomass			Root:shoot ratio		
	df	*F*	*P*	df	*F*	*P*	df	*F*	*P*	df	*F*	*P*
Patch size	1,188	10.8	**0.001**	1,188	1.3	0.259	1,188	10.4	**0.001**	1,188	0.6	0.423
Soil type	1,188	30.8	**<0.001**	1,188	5.8	**0.018**	1,188	31.3	**<0.001**	1,188	0.7	0.416
Species composition	6,188	5.6	**<0.001**	6,188	2.4	**0.030**	6,188	4.8	**<0.001**	6,188	5.3	**<0.001**
Patch size × Soil type	1,188	0.3	0.618	1,188	0.9	0.336	1,188	0.3	0.561	1,188	0.5	0.467
Patch size × Species composition	6,188	0.5	0.813	6,188	0.4	0.897	6,188	0.5	0.820	6,188	0.4	0.900
Soil type × Species composition	6,188	1.6	0.137	6,188	0.7	0.662	6,188	1.4	0.231	6,188	1.8	0.100
Patch size × Soil type × Species composition	6,188	1.5	0.170	6,188	1.5	0.180	6,188	1.5	0.192	6,188	1.7	0.125

**Figure 4.**
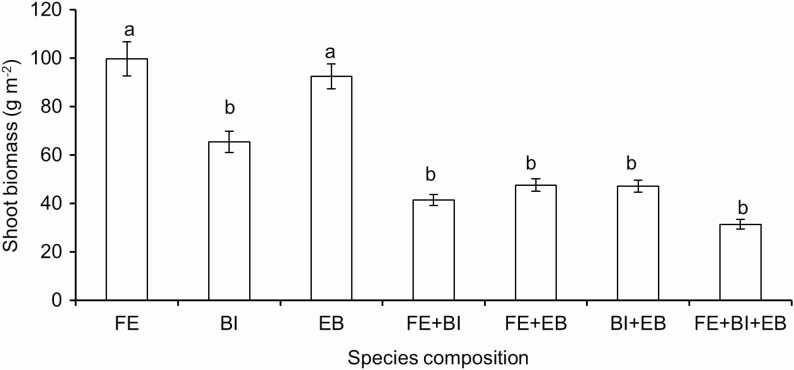
At the community scale, mean ± SE of shoot biomass along species composition, separated by species (i.e. *Festuca elata*, *Bromus inermis* and *Elymus breviaristatus*, labelled as FE, BI and EB, respectively) in pot with all patch sizes. Significant differences between treatments are labelled by different letters (*post hoc* analysis with Bonferroni corrections).

**At the community scale**, species composition significantly affected shoot biomass (*P* < 0.001), where species growing in monoculture had higher shoot biomass, except *B. inermis*, which had lower shoot biomass than *F. elata* and *E. breviaristatus* (Fig. 4). Moreover, another analysis at this scale showed that patch size, soil type, species composition and species significantly influenced shoot biomass (Table 3).

**Table 3.** At the community scale, effects of patch size (medium and large), soil type (nutrient-rich and nutrient-poor substrate), species composition, species and their interactions on shoot biomass of grasses (i.e. *Festuca elata*, *Bromus inermis* and *Elymus breviaristatus*) in MANOVA, where degree of freedom (df), *F*-values and *P*-value are given, and significant results (*P* < 0.05) are labelled in bold. Note that this analysis was done only for pots with medium and large patch as soil type cannot be separated in pots with small patch.

	Shoot biomass		
	df	*F*	*P*
Patch size	1,308	11.9	**0.001**
Soil type	1,308	36.1	**<0.001**
Species composition	6,308	42.0	**<0.001**
Species	2,308	8.0	**<0.001**
Patch size × Soil type	1,308	0.5	0.500
Patch size × Species composition	6,308	1.3	0.241
Patch size × Species	2,308	2.7	0.072
Soil type × Species composition	6,308	0.9	0.470
Soil type × Species	2,308	0.2	0.835
Species composition × Species	3,308	1.6	0.182
Patch size × Soil type ×Species composition	6,308	1.5	0.187
Patch size × Soil type × Species	2,308	0.3	0.746
Patch size × Species composition × Species	3,308	0.4	0.753
Soil type × Species composition × Species	3,308	0.7	0.575
Patch size × Soil type × Species composition × Species	3,308	1.9	0.138

## Discussion

In this study, we found that soil heterogeneity and species composition significantly affected plant biomass and biomass allocation. Specially, with increasing patch size shoot biomass decreased, while root biomass and R:S increased, and total biomass tended to meet a unimodal pattern, where the medium patch had more total biomass.

Our first hypothesis (at the pot scale) expects that more biomass should be in pots with smaller patch, where plants could easier access to resource from neighbouring patches, and such effect may be different among plant species. This is confirmed. We found that species composition significantly affected shoot biomass, root biomass, total biomass and R:S (Table 1). Moreover, shoot biomass (Fig. 2A) decreased with increasing patch size, while root biomass increased with increasing patch size. It is likely due to that plants growing in large patch should grow more roots in order to explore resources from the neighbouring patches (Koch and Matzer 1993; Liu *et al.* 2017a). This is further confirmed by the finding of root:shoot ratio, where it increased with increasing patch size (Fig. 2D). Furthermore, such pattern may be relevant to the activities of soil microbes such as mycorrhizal fungi (Boyrahmadi and Raiesi 2018), which may play different roles in the resource-rich and resource-poor substrates. Reynolds *et al*. (2003) found that microbes could promote plants to coexist by allowing different plant species to access different soil resources. However, the microbes in these two substrates were not measured, which impede us to further test such effects. The opaque nature of soil impedes us to quantify soil heterogeneity. Previous studies mostly focussed on abiotic and biotic factors in soil separately, while Young and Crawford (2004) found that soil-microbe system is self-organized. Thus, future studies on the interactions between abiotic (soil) and biotic (microbe) could show new light on the soil heterogeneity research.

Our second hypothesis (at the substrate scale) assumes that the difference of biomass between nutrient-rich and nutrient-poor substrate should be smaller in smaller patch since plants growing there could uptake resources more easily from their neighbouring patch due to the short distance in smaller patch. This was not supported. The differences of shoot biomass, root biomass, total biomass and R:S between nutrient-rich and nutrient-poor substrates of medium and large patch was not significant **[see****Supporting Information—Appendix Table 1****]**. However, more biomass was indeed found in nutrient-rich than nutrient-poor substrate regardless of patch size, which is consistent with previous studies (Campbell *et al*. 1991; Wijesinghe *et al*. 2001), where plants could apply different strategies to explore resources in nutrient-rich substrates (Caldwell 1994; Fitter 1994; Šmilauerová 2001; Mommer *et al*. 2012). The difference of patch sizes between medium and large patch may not be large enough to distinguish the difference of biomass between nutrient-rich and nutrient-poor substrates, or plants applied here could well adapt to such soil heterogeneity by adjusting their root structure or other traits.

Our third hypothesis (at the community scale) is that more species growing together would support more biomass than growing in monoculture due to the complementarity theory. This is not supported. Plants growing in monoculture had more biomass than growing together with the other species compositions, except *B. inermis* (Fig. 4). This is not consistent with previous studies that diverse species supported higher biomass (Fridley 2001; Loreau and Hector 2001; Bai *et al*. 2004; Ghimire *et al*. 2014; Xi *et al*. 2017). The potential reasons could be that (i) interspecific competition in mixtures was stronger than intraspecific competition in monocultures; or (ii) the belowground competition was stronger in mixtures than in monoculture (Blair 2001); or (iii) plants respond to soil heterogeneity depending on the patch size and the contrast with background soil (Hutchings *et al*. 2003; Rajaniemi 2011). Our results showed that patch size and substrate indeed affected shoot biomass of target plant species in different species composition **[see****Supporting Information—Appendix Fig. 1****]**.

No significant interaction effect of soil heterogeneity and species composition was found at any scale (at the pot, patch or community scale) in this study, which is contrast with the finding of Maestre *et al*. (2006). This is likely caused by the different target species and soil heterogeneity levels between their research and our experiment, where Maestre *et al*. (2006) applied *Lolium perenne*, *Poa pratensis* and *Plantago lanceolata*, which were different with our target plant species (*Festuca elata*, *Bromus inermis*, *Elymus breviaristatus*). Previous studies found that species responded differently to soil heterogeneity (Robinson 1994; Robinson and van Vuuren 1998). Moreover, Maestre *et al*. (2006) developed vertical soil heterogeneity via vertically switching the position of nutrient-rich and nutrient-poor patches, while we created soil heterogeneity by horizontally and vertically altering nutrient-rich and nutrient-poor patches. Plants responded differently to horizontal and vertical soil heterogeneity (Liu *et al*. 2017a, b, 2019), which could be used to explain the different findings between our research and previous studies.

Results of this work should be interpreted and extrapolated with caution as it was done within a short-term, and only a few species was considered. Previous studies found that species number and functional group in grasses-mixture may affect plant biomass (Bergamini *et al*. 2001). Thus, further research on longer-term experiments (Blume-Werry *et al*. 2016; Evans *et al*. 2017) and species that could modify soil heterogeneity such as clonal species (Ying *et al*. 2018) and N-fixing species (Carlsson and Huss-Danell 2003; Bhandari *et al*. 2020) should be considered. Moreover, soil heterogeneity in nature is not with regular patch sizes like in this study, and it tends to be with irregular patches. A series of soil types could be found in natural soil, not only two (nutrient-rich and nutrient-poor) as in our experiment. Thus, simulating such soil heterogeneity with irregular patches sizes and more types of soils may improve our understanding of natural soil heterogeneity.

## Supporting Information

The following additional information is available in the online version of this article—

**Appendix Figure 1.** At the community scale, mean ± SE of shoot biomass along species composition, separated by species (i.e. *Festuca elata*, *Bromus inermis* and *Elymus breviaristatus*, labelled as FE, BI and EB, respectively) in pot with medium and large patch sizes, and small patch is not considered since soil type cannot be separated in this case. Species in the brackets indicates the target species in different composition. Significant differences between treatments are labelled by different letters (*post hoc* analysis with Bonferroni corrections).

**Appendix Table 1.** At the pot scale, effects of patch size (small, medium and large), species composition and their interaction on the differences of shoot biomass, root biomass, total biomass and root:shoot ratio between nutrient-rich and nutrient-poor substrates in MANOVA, where degree of freedom (df), *F*-values and *P*-value are given, and significant results (*P* < 0.05) are labelled in bold. Note that this analysis was conducted for all pots with small, medium and large patch.

plab033_suppl_Supplementary_Materials

## Data Availability

The original data information of figures and tables are available online (https://doi.org/10.5061/dryad.2v6wwpzn9).

## Notes

**Populations & Communities. Chief Editor:** Jean Burns
