# Supplementary material for: Effects of three-dimensional soil heterogeneity and species composition on plant biomass and biomass allocation of grass-mixtures
Source: AoB Plants. 2021 May 28;13(4):plab033. doi: 10.1093/aobpla/plab033 (PMC8255077; doi:10.1093/aobpla/plab033)

**Appendix Table 1** At the pot scale, effects of patch size (small, medium and large), species composition and their interaction on the differences of shoot biomass, root biomass, total biomass and root: shoot ratio between nutrient-rich and nutrient-poor substrates in MANOVA, where degree of freedom (df), *F*-values and *P*-value are given, and significant results (*P* < 0.05) are labelled in bold. Note that this analysis was conducted for all pots with small, medium and large patch

|  | Shoot biomass | | | Root biomass | | | Total biomass | | | | Root: shoot ratio | | | |
| --- | --- | --- | --- | --- | --- | --- | --- | --- | --- | --- | --- | --- | --- | --- |
|  | df | *F* | *P* | df | *F* | *P* | | df | *F* | *P* | | df | *F* | *P* |
| Patch size | 1,94 | 0.2 | 0.636 | 1,94 | 0.7 | 0.414 | | 1,94 | 0.3 | 0.577 | | 1,94 | 0.2 | 0.654 |
| Species composition | 6,94 | 1.5 | 0.193 | 6,94 | 1.0 | 0.411 | | 6,94 | 1.3 | 0.283 | | 6,94 | 1.0 | 0.414 |
| Patch size × Species composition | 6,94 | 1.4 | 0.232 | 6,94 | 1.9 | 0.097 | | 6,94 | 1.4 | 0.241 | | 6,94 | 1.5 | 0.177 |

**Appendix Figure 1** At the community scale, mean ± SE of shoot biomass along species composition, separated by species (i.e. *Festuca elata*, *Bromus inermis* and *Elymus breviaristatus*, labelled as FE, BI and EB, respectively) in pot with medium and large patch sizes, and small patch is not considered since soil type cannot be separated in this case. Species in the brackets indicates the target species in different composition. Significant differences between treatments are labelled by different letters (post-hoc analysis with Bonferroni corrections).


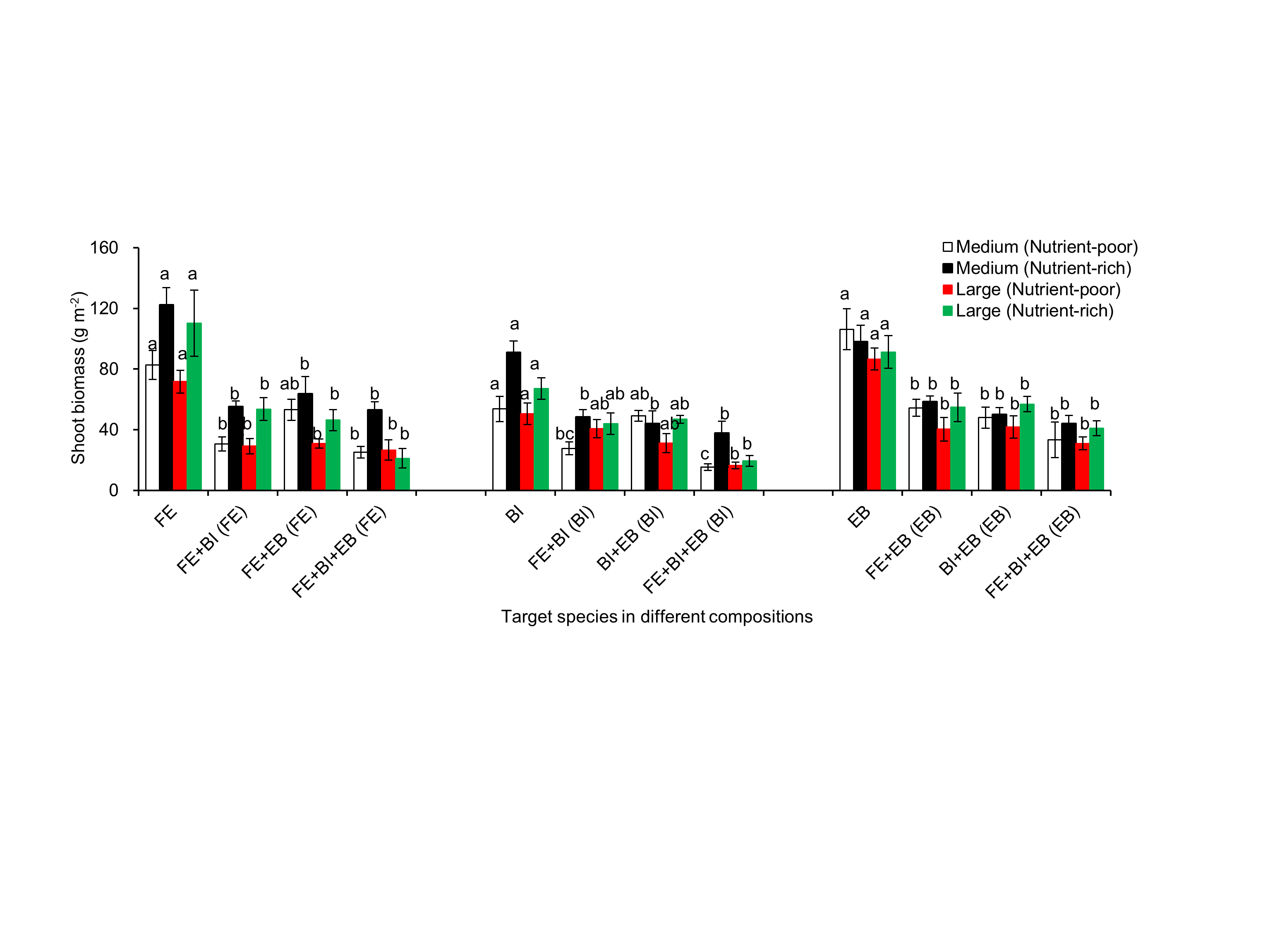

Supplement: plab033_suppl_Supplementary_Materials [file plab033_suppl_supplementary_materials.docx]
